# Extracellular vesicles deliver sodium iodide symporter protein and promote cancer cell radioiodine therapy

**DOI:** 10.1038/s41598-022-15524-9

**Published:** 2022-07-01

**Authors:** Jin Hee Lee, Kyung-Ho Jung, Kim Mina, Kyung-Han Lee

**Affiliations:** 1 Department of Nuclear Medicine, Samsung Medical Center, Sungkyunkwan University School of Medicine, 50 Ilwon-dong, Gangnam-gu, Seoul, Korea; 2 Department of Health Sciences and Technology, SAIHST, Sungkyunkwan University, Seoul, Korea

**Keywords:** Molecular medicine, Oncology

## Abstract

Extracellular vesicles (EVs) are a promising carrier for various cargos with antitumor effects, but their capacity to transfer the ability to transport radioiodine for cancer theranostics remains unexplored. Herein, we tested the hypothesis that EVs can be loaded with the sodium iodide symporter (NIS) protein and efficiently deliver the payload to recipient cancer cells to facilitate radioiodine uptake. The results revealed that donor cells either transduced with an adenoviral vector for transient expression or engineered for stable overexpression secreted EVs that contained substantial amounts of NIS protein but not NIS mRNA. Huh7 liver cancer cells treated with EVs secreted from each of the donor cell types showed significantly increased plasma membrane NIS protein, indicating efficient payload delivery. Furthermore, intact function of the delivered NIS protein was confirmed by significantly increased radioiodine transport in recipient cancer cells that peaked at 48 h. Importantly, NIS protein delivered by EVs significantly enhanced the antitumor effects of ^131^I radiotherapy. These results reveal that EVs are a promising vehicle to deliver NIS protein to cancer cells in sufficient amounts for radioiodine-based theranostics.

## Introduction

Exosomes and extracellular vesicles (EVs) are natural lipid bilayer vesicles inherently able to carry biologic material from donor cells such as mRNA, miRNA, lipids, and proteins^1^. These biomaterial payloads can then be delivered to cancer cells to alter their biologic properties^2–4^. Their physicochemical characteristics offer advantages for clinical application compared to artificial lipid-based liposomes, including low immunogenicity and toxicity, and better tissue penetration for targeting^5^. Therefore, these carriers provide a promising drug-delivery platform for cancer therapy^6–10^.

The search for more precise cancer diagnosis and treatment has led to interest in exploiting exosomes and EVs for tumor theranostics^11,12^. A major theranostic tool is radiopharmaceuticals that deliver radioactive particles to cancer cells for diagnostic imaging as well as to provide targeted radionuclide therapy^13^. Successful theranostic radionuclide therapy is crucially dependent on presence of abundant amounts of target molecules on the malignant cells. Using EVs to deliver target molecules for specific theranostic radionuclide therapy might therefore be a potential anticancer strategy.

A molecule widely exploited for cancer theranostics is the sodium iodide symporter (NIS). This molecule mediates iodine uptake in differentiated thyroid cells and is the basis for radioiodine imaging and therapy of thyroid cancer, which is one of the oldest targets for molecular imaging and targeted radionuclide therapy^14^. Advances in gene therapy methods has extended this strategy to the treatment of non-thyroidal cancers through NIS gene transfer followed by therapeutic radioiodine administration^15,16^. Moreover, the NIS system has advantages over other targeted radionuclide therapies by circumventing limitations including systemic toxicity, immunogenicity, complex radiochemical synthesis, and radioprobe stability. To date, this has relied on gene therapy using viral vectors, which is associated with serious safety issues. In contrast, since EVs are biologically inert and non-toxic, they might provide a safe vehicle for delivering theranostic target molecules to cancer cells.

In this study, we hypothesized that EVs released from transiently or stably transduced donor cells contain NIS protein as payload, and that they are efficiently delivered to recipient cancer cells. We then investigated whether EV-delivered NIS have proper function that can increase iodine transport in magnitudes sufficient to enhance radioiodine therapy in recipient cancer cells.

## Results

### Transduced donor cells efficiently express NIS and show increased radioiodine transport

**Figure 1:**
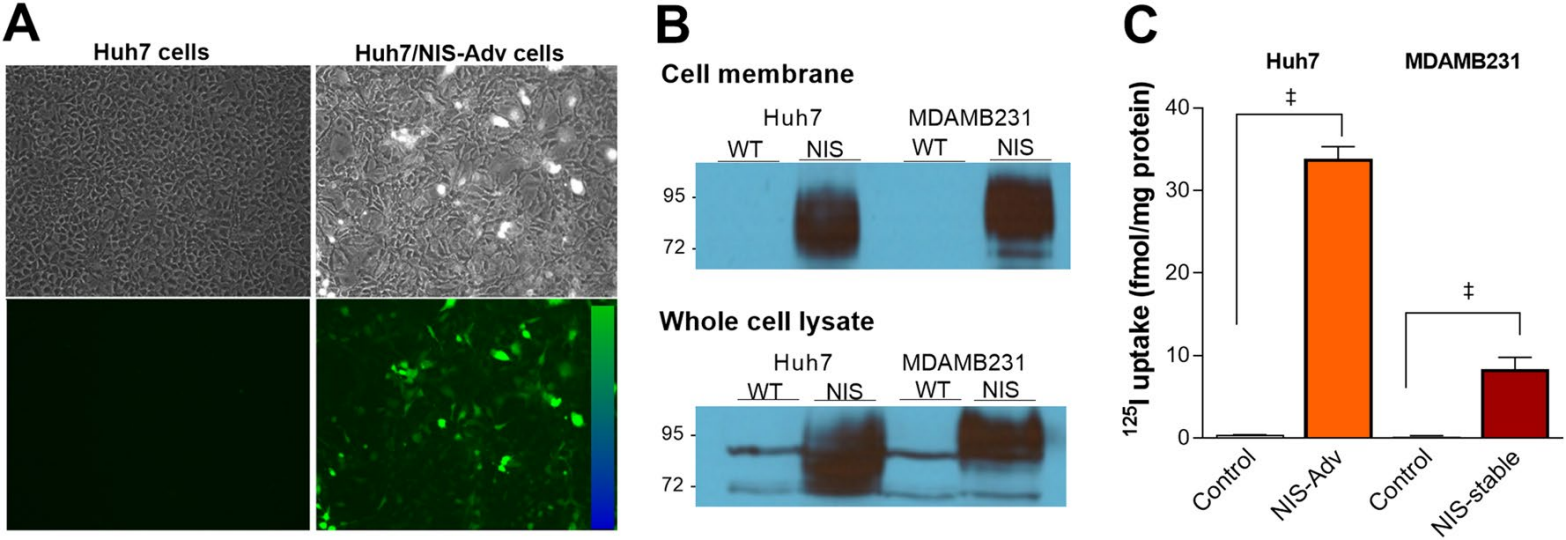
EGFP and NIS proteins in transiently and stably expressing cancer cells. (**A**) EGFP fluorescence in Huh7 cells transduced with NIS/EGFP adenovirus (Huh7/NIS-Adv cells). (**B**) Western blots of NIS protein obtained from the cell membrane fraction (top) and whole cell lysates (bottom) of Huh7, Huh7/NIS-Adv, MDAMB231, and MDAMB/NIS-stable cancer cells. (**C**) ^125^I uptake (right) in Huh7/NIS-Adv and MDAMB/NIS-stable cells compared to respective Huh7 and MDAMB control cells. Bars for uptake are mean ± SD of uptake in fmol/mg protein obtained from triplicate samples per group. *WT* wild type. ^‡^*P* < 0.0001.

Microscopy of Huh7 cells infected with an adenovirus construct containing the enhanced green fluorescent protein (EGFP) gene and human NIS gene (Huh7/NIS-Adv cells) showed high fluorescence at 72 h (Fig. 1a). Western blotting of cell membrane fractions from Huh7/NIS-Adv cells and MDAMB231 cells engineered to stably express NIS (MDAMB231/NIS-stable cells) confirmed strong NIS protein expression, whereas respective control cells did not display visible NIS bands (Fig. 1b).

Furthermore, Huh7/NIS-Adv and MDAMB231/NIS-stable cells showed marked increases of ^125^I uptake that reached 83.2 ± 7.1-fold and 25.2 ± 1.5-fold compared to respective controls (Fig. 1c).

### Characterization of EVs from transient and stable NIS-expressing donor cells

**Figure 2:**
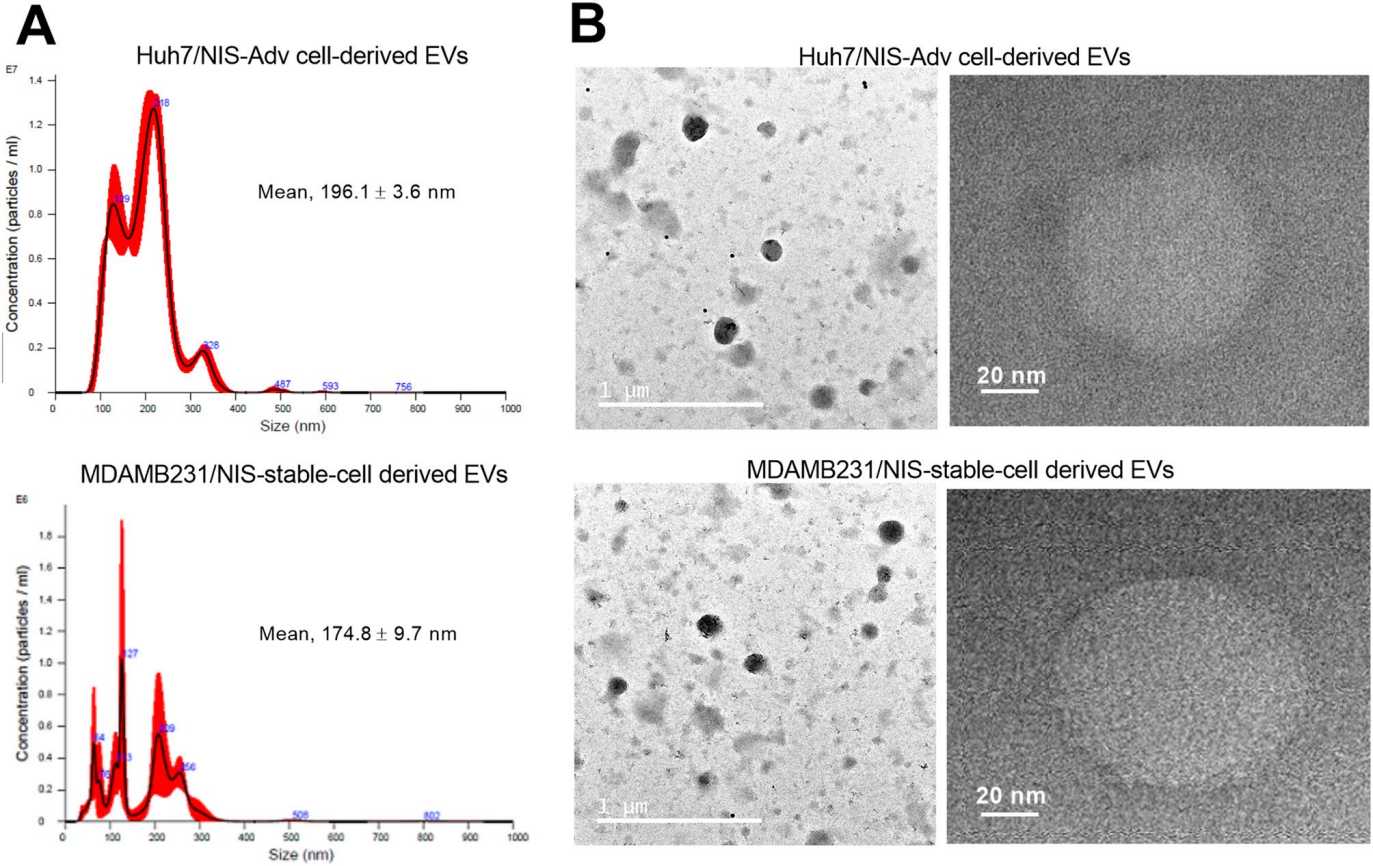
TEM and size distribution of EVs from NIS-expressing cancer cells. (**A**) Nanosight analysis profiles of EVs derived from Huh/NIS-Adv cells (top) and MDAMB231/NIS-stable donor cells (bottom). (**B**) Representative TEM images of EVs derived as above. Magnifications are × 10,000 and × 200,000, and scale bars are 1 μm and 20 nm, respectively.

Malvern Nanosight measurement of our preparation showed mean diameters of 196.1 ± 3.6 and 174.8 ± 9.7 nm for particles derived from Huh7/NIS-Adv cells and MDAMB231/NIS-stable cells, respectively (Fig. 2a). The hydrodynamic profiles displayed several peaks of varying sizes that included those exceeding the 200 nm size limit for exosomes^17,18^.

Transmission electron microscopy (TEM) displayed spherical structures of our preparation derived from Huh7/NIS-Adv and MDAMB231/NIS-stable donor cells (Fig. 2b). On TEM, most of these particles appeared smaller than 200 nm, and there was indication of aggregated particles that could have contributed to the larger sizes on Nanosight measurements. Nonetheless, our particles displayed polydispersity that indicated a mixture of exosomes and EVs and are henceforth referred to as EVs.

### EVs from donor cells contain significant NIS protein but not NIS mRNA

**Figure 3:**
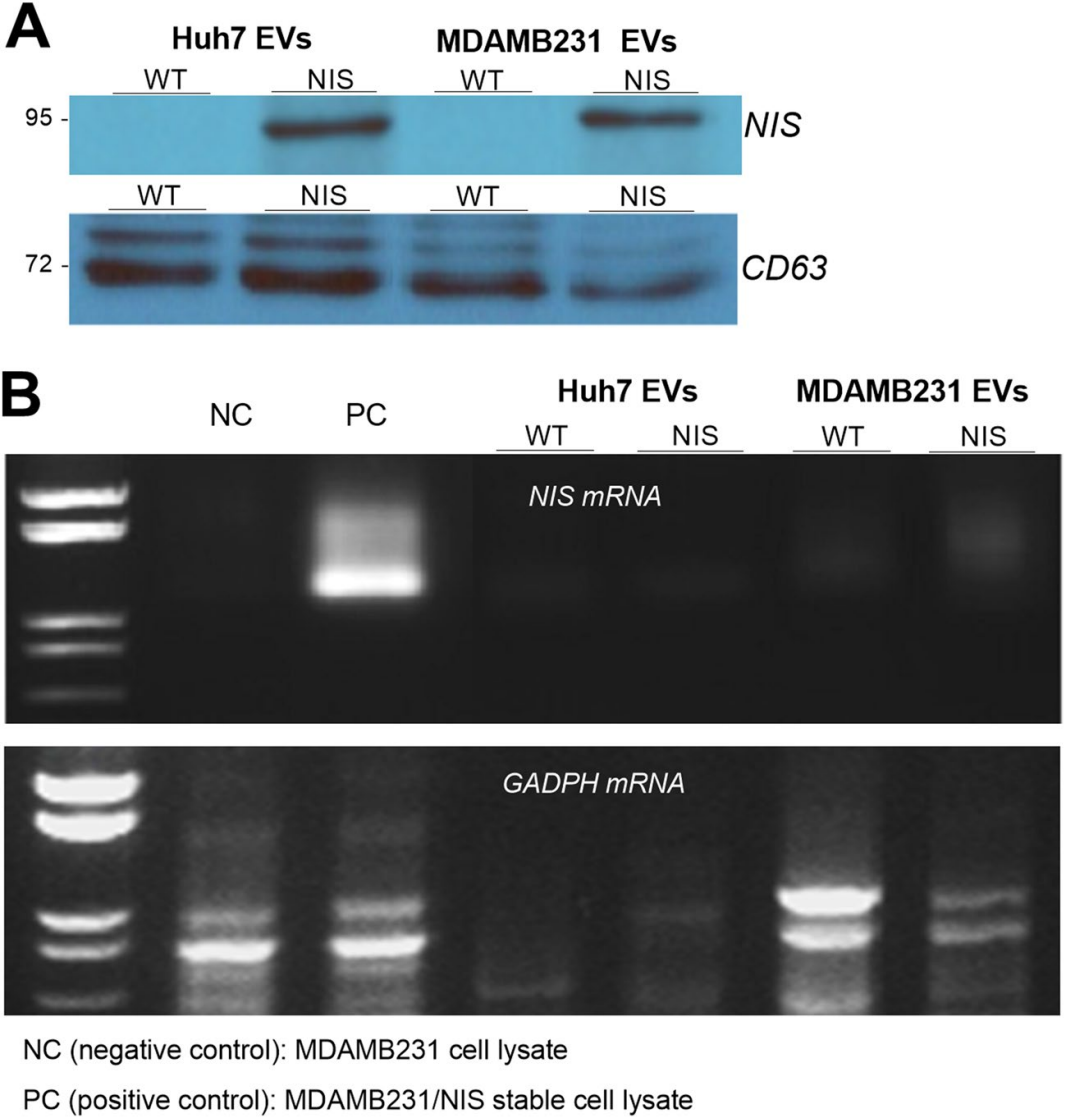
NIS protein and NIS mRNA in EVs from NIS-expressing cancer cells. (**A**) Western blots and quantified band intensities of NIS (top) and CD63 protein (bottom, control) in EVs derived from Huh7, Huh7/NIS-Adv, MDAMB231, and MDAMB/NIS-stable donor cells. (**B**) RT-PCR for NIS mRNA (top) and GADPH mRNA (bottom, control) in EVs from respective donor cells. MDAMB231 and MDAMB231/NIS-stable cells were used for NIS mRNA negative control (NC) and positive control (PC), respectively.

When we investigated the amounts of NIS protein and mRNA carried, Western blots revealed that EVs derived from Huh7/NIS-Adv cells and MDAMB231/NIS-stable cells contained large amounts of NIS protein, whereas those from non-transduced cells did not (Fig. 3a). The exosome marker CD63 was present in high levels in EVs from cells with or without NIS expression (Fig. 3a).

Reverse transcriptase-polymerase chain reaction (RT-PCR) demonstrated high levels of NIS mRNA in MDAMB231/NIS-stable cells that acted as positive control but revealed only trace or undetectable NIS mRNA in EVs of all groups (Fig. 3b). This contrasted with control GADPH mRNA that was present in EVs derived from both NIS gene-transduced and non-transduced cells (Fig. 3b).

### Transfer of NIS protein and iodine transport capacity of recipient cancer cells

**Figure 4:**
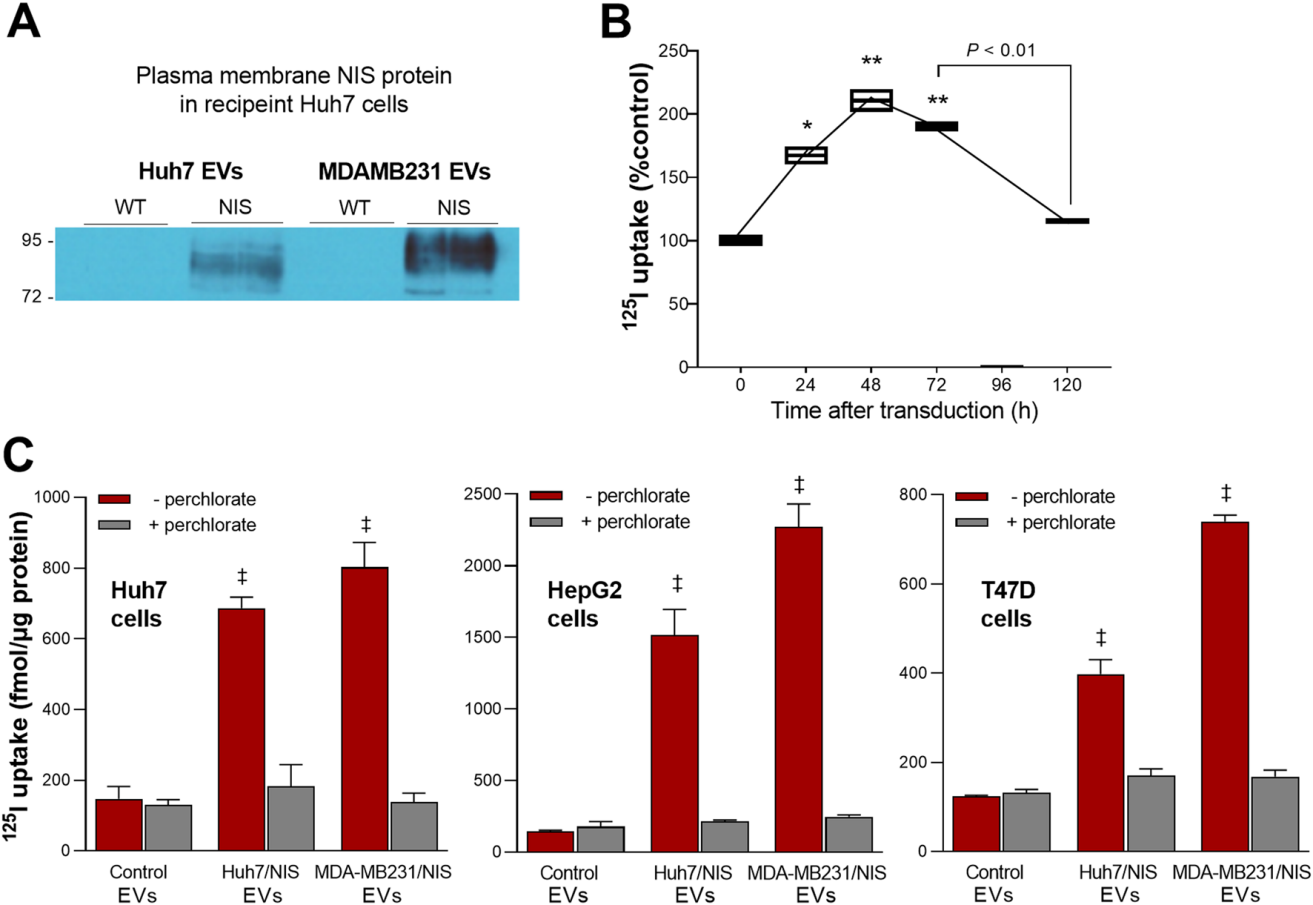
NIS protein and ^125^I uptake in cancer cells treated with EVs. (**A**) Western blots of NIS protein obtained from the plasma membrane fractions of recipient Huh7 liver cancer cells treated with EVs derived from Huh7/NIS-Adv and MDAMB/NIS-stable donor cells (n = 2 per group). (**B**) Time dependence of ^125^I uptake in recipient Huh7 cells treated with EVs s derived from Huh7/NIS-Adv cells. (**C**) ^125^I uptake in recipient Huh7 and HepG2 human liver cancer cells, and T47D human breast cancer cells in 24-well plates at 48 h after treatment with 25 µg per well of EVs derived from Huh7/NIS-Adv or MDAMB/NIS-stable donor cells. All cell uptake data are the mean ± SD of values from triplicate samples per group with or without perchlorate inhibition. *P < 0.05; **P < 0.01; ^‡^*P* < 0.0001, compared to controls.

Recipient Huh7 liver cancer cells treated for 48 h with 100 μg/well (in a 6-well plate) of NIS-containing EVs demonstrated strong NIS protein expression in the cell membrane, indicating efficient NIS delivery and cell surface localization (Fig. 4a).

Radioiodine uptake experiments were performed to verify intact iodine transport function of the NIS protein delivered to recipient cells. We first determined the time course of radioiodine transport capacity in Huh7 cells treated with 25 μg/well (in a 24-well plate) of EVs released from Huh7/NIS-Adv donor cells. The result showed that ^125^I uptake peaked at 48 h post-treatment to 210.4 ± 12.6% of controls and then gradually decreased thereafter (Fig. 4b).

Based on this result, further radioiodine uptake experiments were performed in multiple types of cancer cells in a 24-well plate at 48 h after treatment with 25 μg EVs per well. As a result, EVs from Huh7/NIS-Adv donor cells increased ^125^I uptake of recipient Huh7 and HepG2 human liver cancer cells to 466.3 ± 21.4% and 1095.3 ± 129.1% of respective control levels, and recipient T47D human breast cancer cells to 321.2 ± 26.4% of the control level (Fig. 4c). EVs from MDAMB231/NIS-stable donor cells increased ^125^I uptake of recipient Huh7, HepG2, and T47D cancer cells to 545.4 ± 47.2%, 1641.2 ± 114.7%, and 597.5 ± 11.0% of the control level, respectively (Fig. 4c).

### NIS-loaded EVs enhance the efficacy of ^131^I radiotherapy on recipient cancer cells

**Figure 5:**
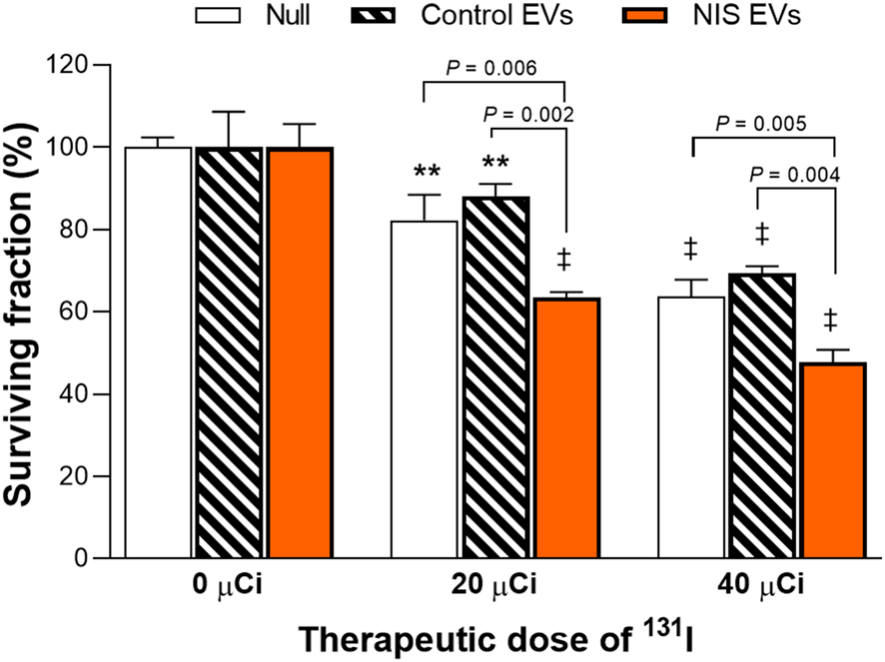
^131^I radiotherapy in cancer cells treated with EVs. Huh7 liver cancer cells were incubated for 48 h with vehicle (null), 10 µg of control EVs, or 10 µg of EVs derived from Huh7/NIS-Adv donor cells. The cells then underwent radiotherapy with 20 or 40 µCi of ^131^I for 7 h and were assessed for survival. Data are mean ± SD of % survival obtained from triplicate samples per group. **P < 0.01; ^‡^*P* < 0.001 compared to untreated controls for each group.

Finally, we determined whether EV delivery of NIS protein can enhance ^131^I radiotherapy in recipient Huh7 cancer cells. In cells without EV pretreatment, 20 μCi ^131^I caused a mild 12.0 ± 3.0% reduction, and 40 μCi ^131^I caused a modest 30.7 ± 1.7% reduction of survival. Pretreatment of cells with control EVs that do not contain NIS protein did not influence ^131^I treatment effect. In contrast, pretreatment with EVs from Huh7/NIS-Adv donor cells enhanced the antitumor effects of 20 and 40 μCi of ^131^I, causing further reductions of recipient cell survival to 36.5 ± 1.2% and 52.3 ± 3.0%, respectively (both *P* < 0.005 compared to cells without EV treatment; Fig. 5).

## Discussion

This study confirms that EV released by donor cells transiently transduced or engineered for stable overexpression contain abundant amounts of NIS protein. The study further reveals that treatment of cancer cells with these EVs efficiently delivers NIS protein to the recipient cells and transfers the ability to avidly transport radioiodine.

Exosomes and EVs contain a huge variety of proteins^19^, which include not only exosome biomarkers such as CD63 and caveolin-1^20^, but also membrane proteins and integrins that play important roles in cancer biology^21,22^. Thus, EV-mediated transfer of specific proteins might provide a unique opportunity for cancer theranostics, provided that a large amount of the therapeutic protein is loaded as cargo^9^. In this study, we showed that EVs secreted by donor cells transiently or stably transduced for NIS overexpression contained substantial quantities of NIS protein but not NIS mRNA. Thus, whereas therapeutic loads previously investigated have mostly consisted of genetic materials^23–25^, NIS protein is included as an EV payload for potential cancer therapy.

We next investigated whether EV-loaded NIS protein is effectively transferred to cancer cells that have low baseline NIS expression. As a result, Western blotting of NIS-expressing cells, EVs derived from these cells, and recipient cells treated with the EVs showed distinct NIS protein bands at approximately 90 kDa, the expected size for fully glycosylated NIS. Furthermore, the recipient cells, like donor cells, showed strong NIS protein expression in the plasma membrane. These results are consistent with export of EV-transferred NIS to the cell surface, where they can mediate radioiodine uptake.

It is also important to verify that the transferred NIS protein retains normal iodide transport function in recipient cells. Membrane receptors delivered via exosomes have been shown to integrate into the plasma membrane^22,26^ and exert normal chemo-resistance activity^26^. In our results, NIS protein transfer substantially increased radioiodine uptake as high as 16-fold of the baseline amount in control cancer cells that had undetectable intrinsic NIS expression. This confirms normal transport function of exosome-delivered NIS protein in recipient cancer cells.

Time course experiments revealed that increase of radioiodine uptake peaked at 48 h following EV treatment and then gradually decreased thereafter. This indicates gradual degradation of the delivered NIS protein in recipient cells and coincides with the known 3-day half-life of NIS protein in the absence of thyroid stimulating hormone stimulation^27^. The timeline of enhanced iodide transport activity following protein delivery will need to be considered when designing theranostic applications of exosome NIS.

Importantly, we also explored the antitumor effect of ^131^I radionuclide therapy following EV NIS delivery. The results revealed significantly greater suppression of recipient Huh7 cancer cell survival by graded ^131^I doses following treatment with NIS-loaded EVs but not control EVs. This supports the potential usefulness of EVs as an NIS protein delivery system for cancer therapy.

In this study, we used cancer cell-derived EV because cancer cells that produce substantially greater amounts compared to non-tumor cells. Obtaining greater amounts of EVs helped facilitate our study that required transfer of large NIS protein that have lower loading capacity compared to small molecules. Cancer cell-derived exosomes show therapeutic potential in cancer disease^28,29^, but their clinical use of can raise concerns of adverse effects. Therefore, our results will need to be verified by future investigations using EVs from non-tumor donor cells.

Our in vitro findings will need to be verified through in vivo animal experiments that include confirmation of sufficient tumor retainment of radioiodine. These experiments were difficult to perform in the present study because of the requirement of large-scale EV preparation. In the future, surface functionalization for target-specific delivery^30^ may help facilitate the in vivo translation of NIS EV therapy.

## Conclusion

EVs can be loaded with NIS protein that are efficiently delivered as payload to recipient cancer cells. Furthermore, the delivered NIS protein transfers increased radioiodine transport function to recipient cancer cells, enhancing the antitumor effect of radioiodine therapy. EVs thus provide a biologically safe carrier for NIS protein delivery to cancer cells for radionuclide theranostics.

## Materials and methods

### Cell culture

Huh7 and HepG2 human liver cancer cells and MDAMB231 and T47D human breast cancer cells were from the American Type Culture Collection. Cells were maintained in high-glucose DMEM (Huh7 cells and MDAMB231), EMEM (HepG2 cells), or RPMI (T47D cells) supplemented with 10% fetal bovine serum (FBS; Serena, Germany) and 1% penicillin/streptomycin (Gibco Laboratories) at 37 °C and 5% CO2 in a humidified atmosphere. The cells were confirmed mycoplasma free and authenticated by our institutional research support center.

Huh7 cells were transiently transduced to express NIS (Huh7/NIS-Adv cells) by infection with a replication deficient human recombinant adenoviral construct containing the human NIS gene along with the EGFP gene (NIS/EGFP adenovirus)^18^. Following incubation for 8 h with the adenovirus, cells were carefully washed twice with phosphate-buffered saline (PBS) and fresh culture medium was added. EVs were obtained over 72 h for subsequent experiments.

MDAMB231 cells engineered to stably express NIS (MDAMB231/NIS-stable cells) were kindly provided by Dr. Youn H (Seoul National University College of Medicine, Korea). The cells were sub-cultured 2 times a week and used at passages less than 10.

### EV isolation

For EV purification, cells were cultured for at least 72 h in exosome-depleted FBS. Culture media underwent a differential centrifugation protocol of sequential steps at 500*g* for 10 min, 2000*g* for 10 min, and 10,000*g* for 30 min to remove cell debris for EV isolation. This resulting supernatant was ultra-centrifuged at 100,000*g* for 2 h at 4 °C (Beckman Coulter), and the pellet was suspended in PBS and ultra-centrifuged at 100,000*g* for another 2 h. EV content was determined by BCA protein assays (Thermo Scientific) after lysis with RIPA buffer (Sigma-Aldrich).

### Size and concentration measurements and transmission electron microscopy (TEM)

For size and concentration measurements, 5–10 μg of EVs were resuspended in 1 mL of PBS and measured with a NanoSight NS300 instrument (Malvern, UK).

For TEM, 10 µL (100 µg/mL) of purified EVs were placed on non-glow-discharged carbon-coated Formvar/Carbon grids (PolySciences) and negatively stained for 1 min with 10 µL of 2% osmium tetroxide solution (Sigma-Aldrich). Negative stain solution was removed by wicking onto filter paper, and TEM images were obtained using a JEM-1400 Flash transmission electron microscope (Jeol, Japan).

### Cellular radioiodine uptake measurement

Monolayer cells in 24-well plate were incubated for 1 h with 74 kBq of ^125^I (Perkin Elmer, MA) added to the culture medium in 5% CO_2_ at 37 °C. Cells were rapidly washed twice with cold PBS, lysed with 0.1 N NaOH, and measured for cell-bound radioactivity on a γ-counter (Wallac). ^125^I uptake levels were normalized to the cellular protein content by Bradford assay (Bio-Rad Laboratories, CA) and expressed in fmol per mg or μg of protein.

### Immunoblotting for CD63 and plasma membrane NIS protein

To prepare plasma membrane protein, cells were washed twice with cold PBS. After 15 min incubation in ice with 0.5 mL of solution-A containing sucrose, 1 mM EDTA, 1 mM phenylmethylsulfonyl fluoride, and aprotinin in 10 mM HEPES, the cells were lysed by sonication (10 times) and centrifuged at 14,000 rpm for 10 min at 4 °C. The supernatant was collected, mixed with 50 µL of 1 M Na_2_CO_3_, and incubated in ice for 1 h. The mixture was then transferred to a Beckman tube that was filled with solution-B containing sucrose and 1 mM MgCl_2_ in 10 mM HEPES and ultracentrifuged at 42,000 rpm for 1 h at 4 °C. The supernatant was decanted, and the membrane fraction pellet was finally resuspended in distilled water. To obtain total cellular protein, cells were washed and lysed with a cold protein extraction solution (PRO-PREP; Intron, Korea) that contained a protease inhibitor cocktail (Sigma-Aldrich). EV protein was prepared by lysis with RIPA buffer.

Plasma membrane and total cellular protein (20 μg) or EV protein (5 μg) were separated by electrophoresis on a 10% sodium dodecyl sulfate polyacrylamide gel, followed by transfer to a polyvinylidene difluoride membrane. The membrane was blocked with 5% nonfat milk in Tris-buffered saline and 0.5% tween-20 (TBST) for 1 h at room temperature and incubated overnight at 4 °C with a rabbit antibody against human NIS (Imanis, MN; #REA004) or mouse antibody against human CD63 (Invitrogen, #10628D). After washing with TBST, the membranes were incubated for 1 h at room temperature with a secondary anti-rabbit IgG (Cell Signaling, #7074) for NIS and an anti-mouse IgG (Cell Signaling, #7076) for CD63. Immune reactive proteins were detected by chemiluminescence (Thermo Scientific), and band intensities were quantified on a GS-800 densitometer using Quantity One software (Bio-Rad Laboratories, CA).

### Reverse transcriptase-polymerase chain reaction (RT-PCR) for NIS mRNA

RNA was extracted from cells or EVs with the Quick RNA micro prep kit (Zymo). RNA was quantified, and 1 μg underwent RT-PCR for NIS mRNA or GADPH mRNA using AccuPower® RT-PCR PreMix (Bioneer, Daejeon, Korea) according to the manufacturers protocol. The thermal cycle profile was denaturation at 95 °C for 3 min and 35 cycles of 94 °C for 1 min and 60 °C for 30 s, followed by extension at 72 °C for 1 min using specific primers. The primer sets used for NIS mRNA were forward, 5'-CCATCCTGGATGACAACTTGG-3' and reverse, 5'-AAAAACAGACGATCCTCATTGGT-3'. Those for GADPH mRNA were forward, 5'-GAGCCCGCAGCCTCCCGCTT-3' and reverse, 5'-CCCGCGGCCATCACGCCACAG-3'.

### In vitro ^131^I therapy and sulforhodamine B (SRB) survival assay

Huh7 cells on a 96-well plate were treated with 10 μg of EVs per well for 48 h. After 48 h, 20 μCi or 40 μCi of ^131^I was added to the medium in each well, and the medium was replaced with fresh medium after 7 h of incubation at 37 °C. ^131^I therapy involved another 70 h of incubation at 37 °C.

Surviving cell content was measured by SRB assays. Briefly, cells were fixed with 10% (wt/vol) trichloroacetic acid at 4 °C and stained with SRB dye (Sigma-Aldrich) for 30 min. Excess dye was removed by repeated washing with 1% (v/v) acetic acid. Protein-bound dye was eventually dissolved in 10 mM tris base solution and subjected to spectrophotometric measurement of absorbance at 510 nm using a VERSA max microplate reader (Molecular Devices, LLC).

### Statistical analysis

Statistical analyses were carried out using the two-tailed unpaired Student’s *t*-test. Data are represented as mean ± standard deviation (SD). *P* values < 0.05 were considered statistically significant.

## Supplementary Information

Supplementary Information.

## Data Availability

All data generated or analyzed during this study are included in this published article.
