# Supplementary material for: Extracellular vesicles deliver sodium iodide symporter protein and promote cancer cell radioiodine therapy
Source: Sci Rep. 2022 Jul 1;12:11190. doi: 10.1038/s41598-022-15524-9 (PMC9249836; doi:10.1038/s41598-022-15524-9)
Supplement: Supplementary file 1 — Supplementary Information. [file 41598_2022_15524_MOESM1_ESM.docx]

**Supplementary information (full unedited X-ray films and gels)**

**Extracellular Vesicles Deliver Sodium Iodide Symporter**

**Protein and Promote Cancer Cell Radioiodine Therapy**

(Short title: **NIS Extracellular Vesicles for I-131 Cancer Therapy**)

Jin Hee Lee, PhD*^1,2^*, Kyung-Ho Jung, PhD*^1,2^*, Kim Mina, Kyung-Han Lee, MD*^1,2*^*.

^1^Department of Nuclear Medicine, Samsung Medical Center, Sungkyunkwan University School of Medicine; ^2^Department of Health Sciences and Technology, SAIHST, Sungkyunkwan University, Seoul, Korea

***Corresponding Author (for reprints):** Kyung-Han Lee, MD, PhD.

Nuclear Medicine, Samsung Medical Center, 50 Ilwon-dong, Gangnam-gu, Seoul, Korea

Tel: 82-2-3410-2630; Fax: 82-2-3410-2639; [khleenm@naver.com](mailto:khleenm@naver.com)

**First Author:** Jin Hee Lee, PhD.

Address as above; Tel: 82-2-3410-2649; Fax : 82-2-3410-2639; [mapcar@hanmail.net](mailto:mapcar@hanmail.net)

This research was supported by the Basic Science Research Program through the National Research Foundation of Korea (NRF) funded by the Ministry of Science, ICT, and Future Planning (2019R1A2C2007455).

**Full unedited gel for Figure 1B**


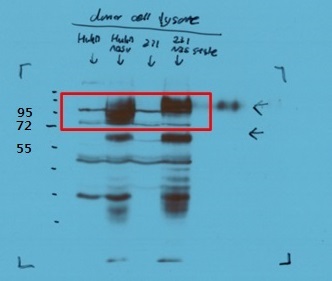


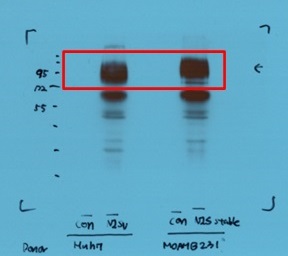


**Supplementary Figure 1:** Full-length Western blots of protein from membrane fraction (left) and whole lysate (right) for detection of NIS in Fig-1B.

* Please note that X-ray films were cut into required sizes with a paper cut knife before use for exposure. They are therefore shown as small sized films with right-angled edges.

**Full unedited gel for Figure 3A**


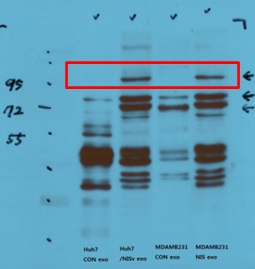


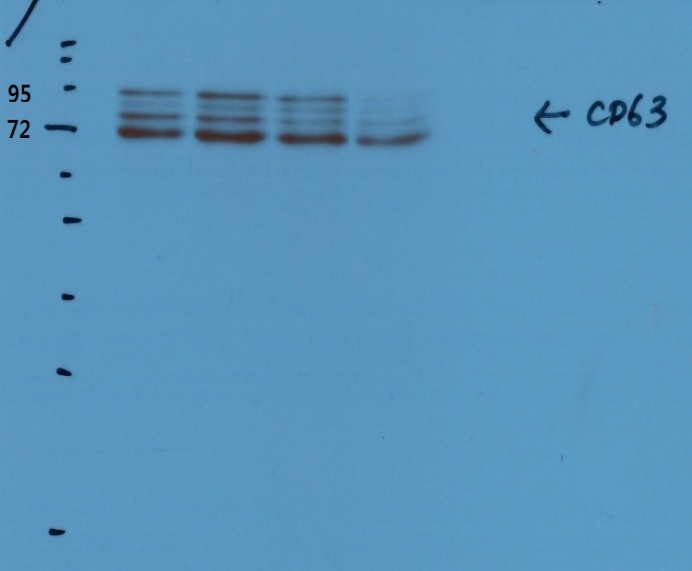


**Supplementary Figure 2:** Full-length Western blots of protein from EVs for detection of NIS (left) and CD63 (right) for Fig-3A.

* Please note that X-ray films were cut into required sizes with a paper cut knife before use for exposure. They are therefore shown as small sized films with right-angled edges.

**Full unedited gel for figure 3 B**


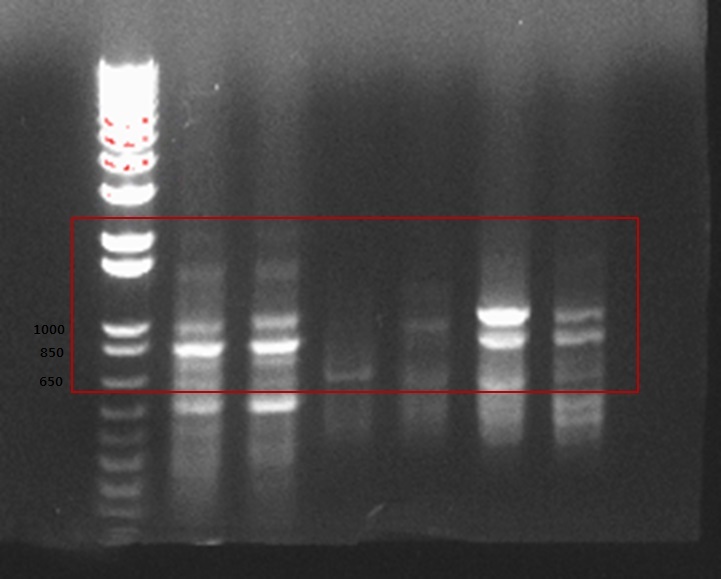

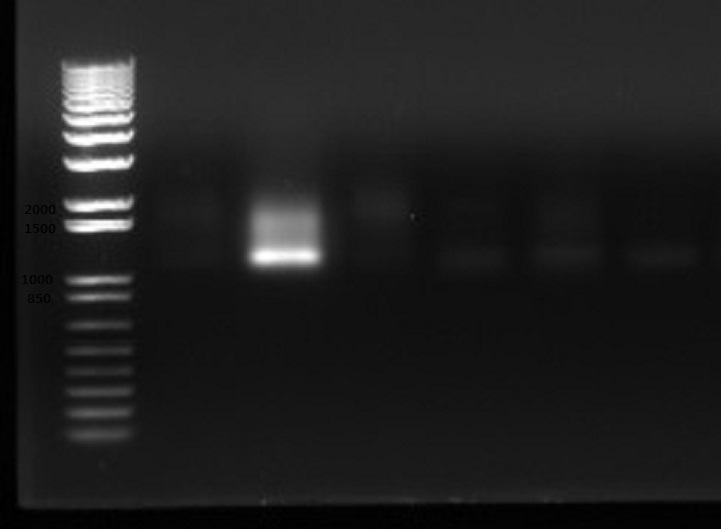


**Supplementary Figure 3:** Full-length gels of RT-PCR of mRNA from EVs for detection of NIS mRNA (left) and GAPDH mRNA (right) in Fig-3B.

**Full unedited gel for Figure 4A**


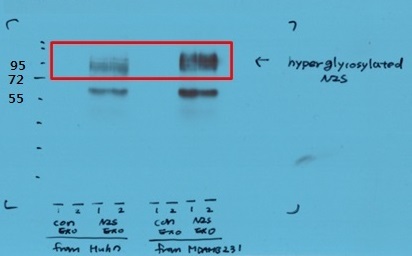


**Supplementary Figure 4:** Full-length Western blots of protein from membrane fraction for detection of NIS in Fig-4A.

* Please note that the X-ray film was cut into the required size with a paper cut knife before use for exposure. It is therefore shown as a small sized film with right-angled edges.
